# Supplementary material for: Insc:LGN tetramers promote asymmetric divisions of mammary stem cells
Source: Nat Commun. 2018 Mar 9;9:1025. doi: 10.1038/s41467-018-03343-4 (PMC5844954; doi:10.1038/s41467-018-03343-4)
Supplement: Supplementary file 1 — Supplementary Information [file 41467_2018_3343_MOESM1_ESM.pdf]

**Insc:LGN tetramers promote asymmetric divisions of mammary stem cells**

Culurgioni et al.

**Supplementary Movie 1**. Animated summary of the close-up views at the interface between subunits of the $dLGN^{TPR}$:$Insc^{ASYM}$ tetramer, as presented in Fig. 1-2-3 and Supplementary Figure 3.

**Supplementary Movie 2.** Morphing between the extended and compact conformers of the $dLGN^{TPR}$:$Insc^{ASYM}$ tetramer present in the crystallographic a.s.u.

## SUPPLEMENTARY INFORMATION

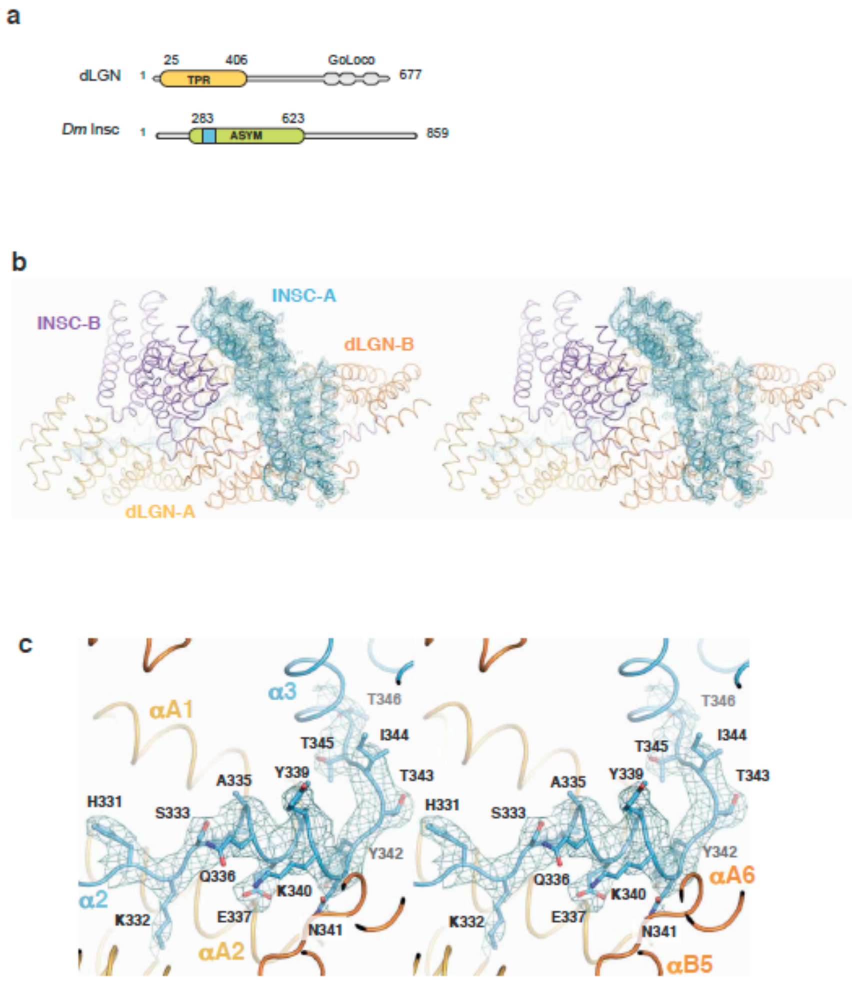


**Supplementary Figure 1. (a) Domain organization of *Drosophila* dLGN and Insc**. The numbers indicate the boundaries of the TPR domain of dLGN and the Asymmetric domain of Insc used for structure determination. The Insc stretch corresponding to the Insc$^{PEPT}$ sufficient for the high affinity interaction with dLGN$^{TPR}$ is colored in cyan. (**b-c**) Stereo views of final electron density maps. The presented 2mFo-DFc maps for the entire Insc-1 chain, or around helix α2-α3 of Insc-1 are contoured at 1.5 σ and 1.0 σ level, respectively.

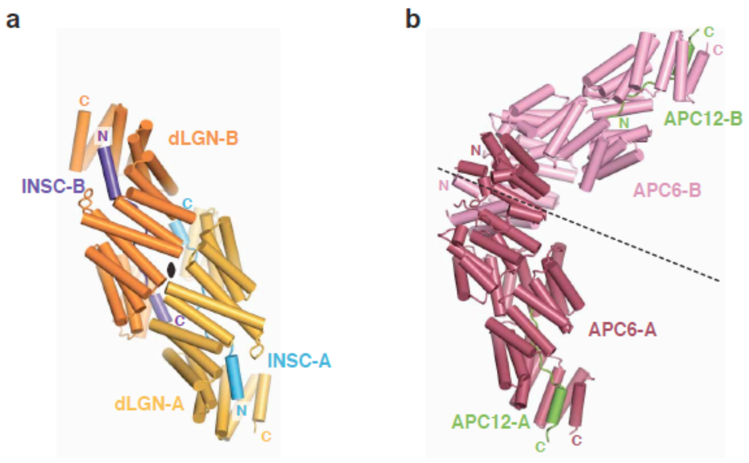


**Supplementary Figure 2. Comparative views of the TPR homodimers of dLGN and APC6.** (**a**) The TPR domains of dLGN form head-to-head interactions induced by the Insc chains, which runs in opposite direction compared to dLGN. The two-fold axis running between the TPR4-5 of the two subunits is marked with a black oval. For clearness, only the N-terminal Insc$^{PEPT}$ stretch of Insc$^{ASYM}$ is shown in the figure. The tetramer subunits are colored as in Fig 1. (**b**) V-shaped arrangement of TPR subunits APC6 of the Anaphase Promoting Complex (PDB ID 2XPI). APC6 molecules assemble in homotypic dimers mediated by extensive interaction of the N-terminal TPR repeats. The dimer two-fold axis is depicted as a dashed black line. APC12 subunits recognize the inner groove of the C-terminal TPR units of APC6 with a topology reminiscent of the dLGN:Insc complex, although with an opposite chain directionality with respect to the TPR domain.

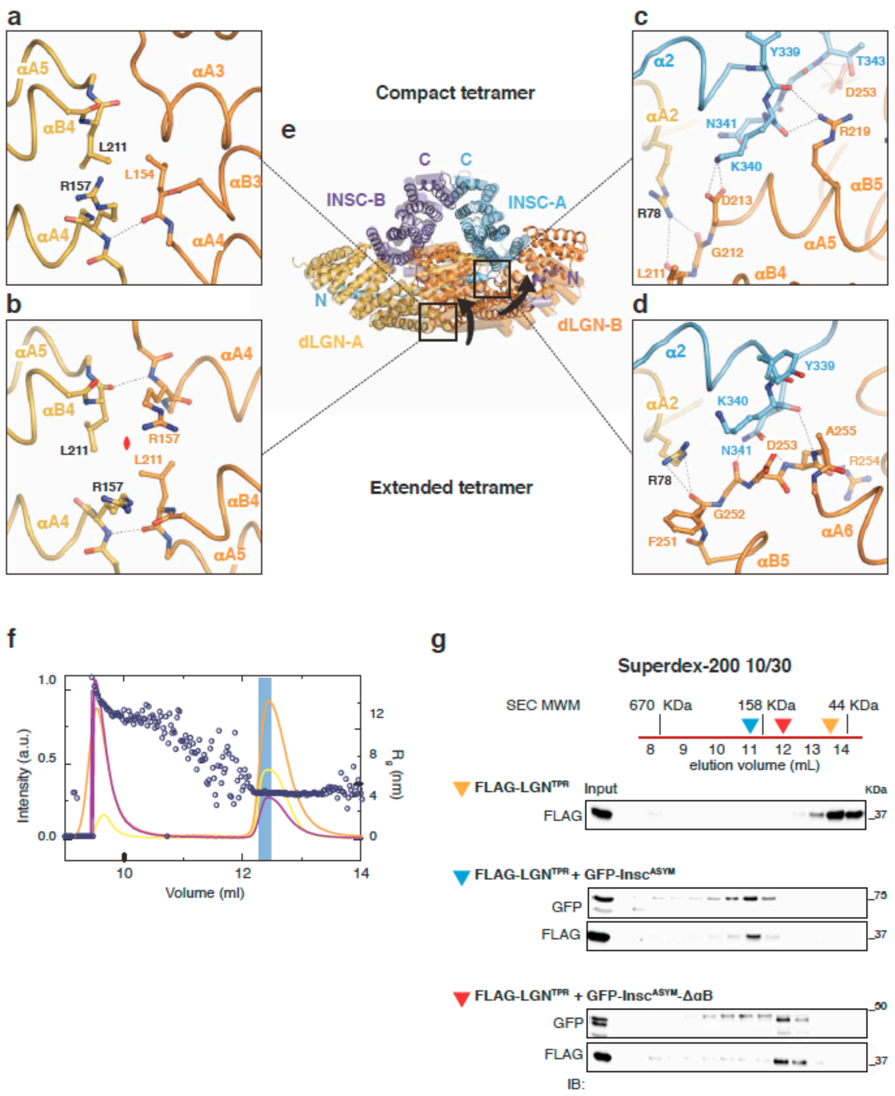


**Supplementary Figure 3. Rearrangement of the TPR-TPR interface in the extended and compact conformation of the tetramers.** (**a-b**) Ribbon representation of the residues of dLGN-*A* (gold) facing TPR3 or TPR4 of dLGN-*B* (orange). In the extended conformation the TPR4 of the two dLGN subunits organize a symmetrical interface contributed by equivalent hydrogen bonds between main-chain atoms of Leu211 and Arg157 (bottom). A red oval marks the position of the 2-fold symmetry axis passing through TPR4-5 of dLGN-*A* and dLGN-*B*. The reciprocal movement of the two TPR domains observed in the compact conformers induces a frameshift of one TPR repeat at the TPR4 interface (top). As a result, TPR4 of dLGN-*A* is positioned in front of TPR3 of dLGN-*B*, and the backbone nitrogen of Arg157 engages in a hydrogen bond with the carbonyl of Leu154. (**c-d**) Topology of the

interface between helix α2 of Insc-1 and the TPR domains of dLGN-*A* and dLGN-*B* in the two tetrameric conformations. In the extended conformer the last turns of helix α2 of Insc-1 are hydrogen-bonded with the loop connecting TPR5 and TPR6 of dLGN-*B* (bottom), which readapt to bind the loop between TPR4-5 in the compact conformation (top). Overall, the plasticity of the interface between subunits implies a major topological robustness of the tetramer. (**e**) Superposition of the extended and compact tetramers depicted as in Fig. 2a with the position of the close-up views indicated in squares. (**f**) SEC-SAXS chromatogram of $dLGN^{TPR}$:$Insc^{ASYM}$ complex with UV-absorbance curve (yellow), total scattering intensity (orange), forward scattering intensity (violet) and radius of gyration (blue open circles). Data frames used to calculate the one-dimensional scattering curve of Fig. 2e are highlighted in light blue. (**g**) $Insc^{ASYM}$-ΔαB forms 1:1 complexes with $LGN^{TPR}$ according to SEC analysis. SDS-PAGE separation and WB analysis of peak fractions of Superdex-200 10/30 runs of HEK293T cell lysates transiently transfected with plasmids expressing FLAG-$LGN^{TPR}$ alone (orange triangle) or in combination with GFP-$Insc^{ASYM}$ (blue triangle) or GFP-$Insc^{ASYM}$-ΔαB (red triangle). $LGN^{TPR}$ in isolation elutes as a monomeric species at about 44 KDa (top), and it co-elutes with $Insc^{ASYM}$ slightly before the 158 KDa Molecular Weight Marker (middle), as expected for a 2:2 complex. $LGN^{TPR}$ and $Insc^{ASYM}$-ΔαB coelute between the 158 KDa and the 44 KDa MWM (bottom) indicating that the form a lower-stoichiometry complex, compatible with a 1:1 assembly. We noticed that the anti-GFP immunoblot of the $Insc^{ASYM}$-ΔαB sample revealed a slight degradation at the C-terminus of $Insc^{ASYM}$-ΔαB, that might correspond to the loss of one helix of the ARM3.

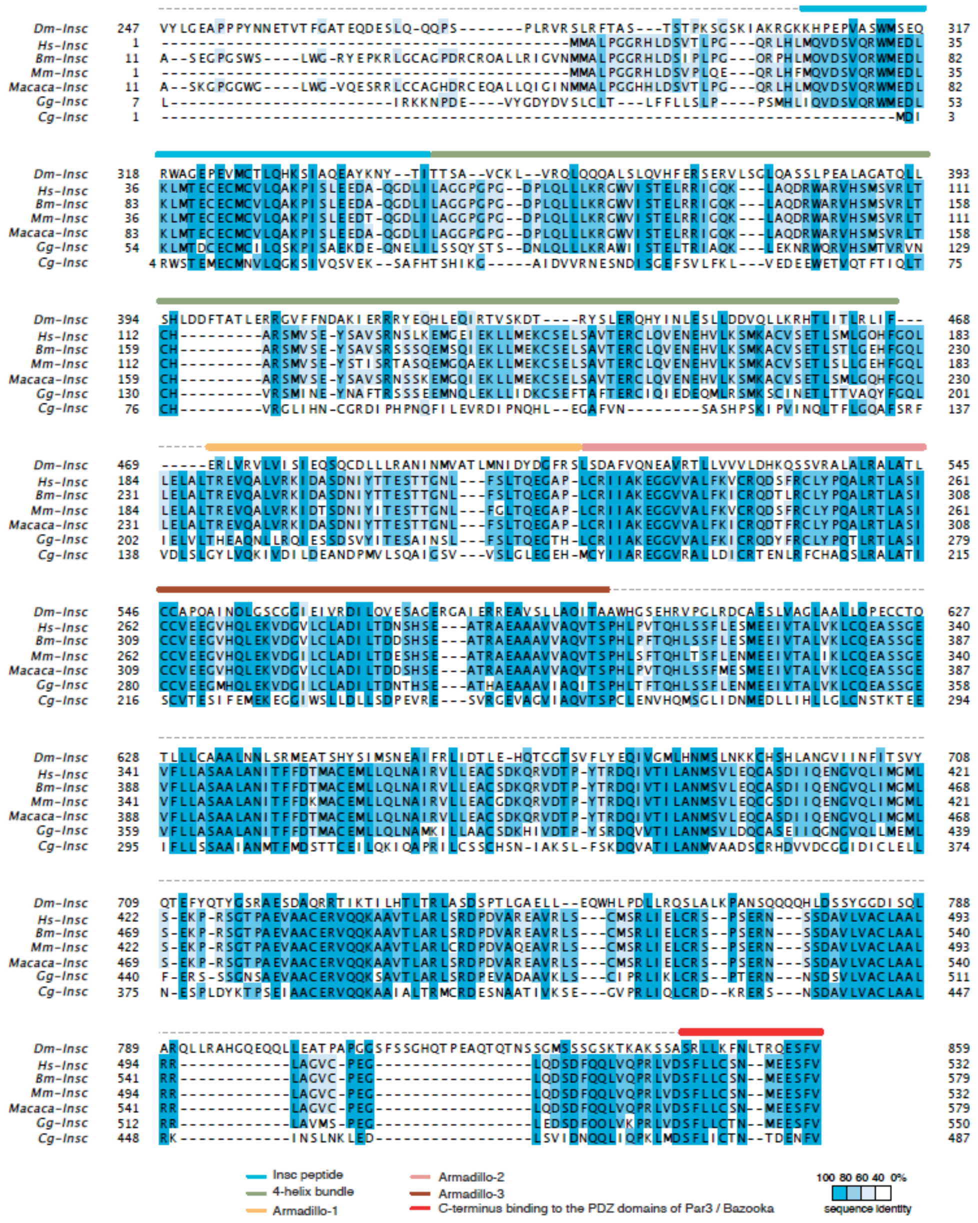

**Supplementary Figure 4. Sequence alignment of Insc orthologues.** Insc residues are colored according to their conservation calculated on the basis of the alignment of seven orthologues from *Drosophila melanogaster, Homo sapiens, Bos mutus, Mus musculus, Macaca mulatta, Gallus gallus,* and *Crassostrea gigas*. The boundaries of the structural motifs of Insc$^{ASYM}$ are displayed on top of the sequence with the same color code of Fig. 3, and the C-terminal tail important for the binding to Par3 / Bazooka is labeled in red. Region of Insc for which no structural information is available are indicated in dashed gray lines.

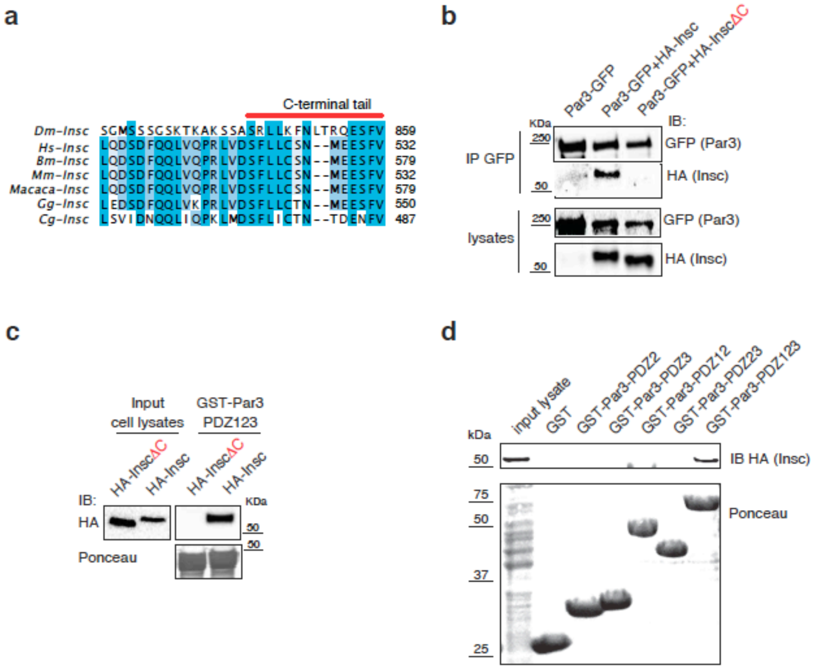


**Supplementary Figure 5. Binding of Insc to the PDZ123 portion of Par3.** (**a**) Sequence alignment of the C-terminal tail of Insc colored according to conservation as in Fig 3. (**b**) Interaction of Par3 with human Insc. HEK293T cells were transiently transfected with plasmids containing human Par3-GFP alone or in combination with HA-tagged human Insc or InscΔC (lacking the last 16 residues). After 24 hours, cells were treated with 0.33 mM nocodazole for 16 hours. Cell lysates were immunoprecipitates (IPs) with anti-GFP antibodies conjugated to sepharose beads, and immunoblotted (IB) with the indicated antibodies. Only full-length Insc was able to bind Par3. (**c**) Interaction of the PDZ domain of human Par3 with full-length Insc. GST-Par3$^{PDZ123}$ was immobilized on beads and incubated with nocodazole-treated HEK293T lysates overexpressing HA-Insc, full-length or C-terminally truncated. After washes, species retained on beads were analysed by IB. Only full-length Insc and not a C-terminally truncated construct can interact with purified GST-Par3$^{PDZ123}$ adsorbed on beads. (**d**) GST-fusion proteins containing several combinations of the three PDZ domains of Par3 were adsorbed on glutathione beads, and incubated with HEK293T lysates transfected with HA-Insc. The fusion GST-Par3-PDZ1 was omitted because the sample was poorly soluble. After washes, species retained on beads were analysed by SDS-PAGE and anti-HA

immunoblotting (IB). The Ponceau staining of the blotted membrane is shown as a loading control. Insc associates only with Par3-PDZ123.

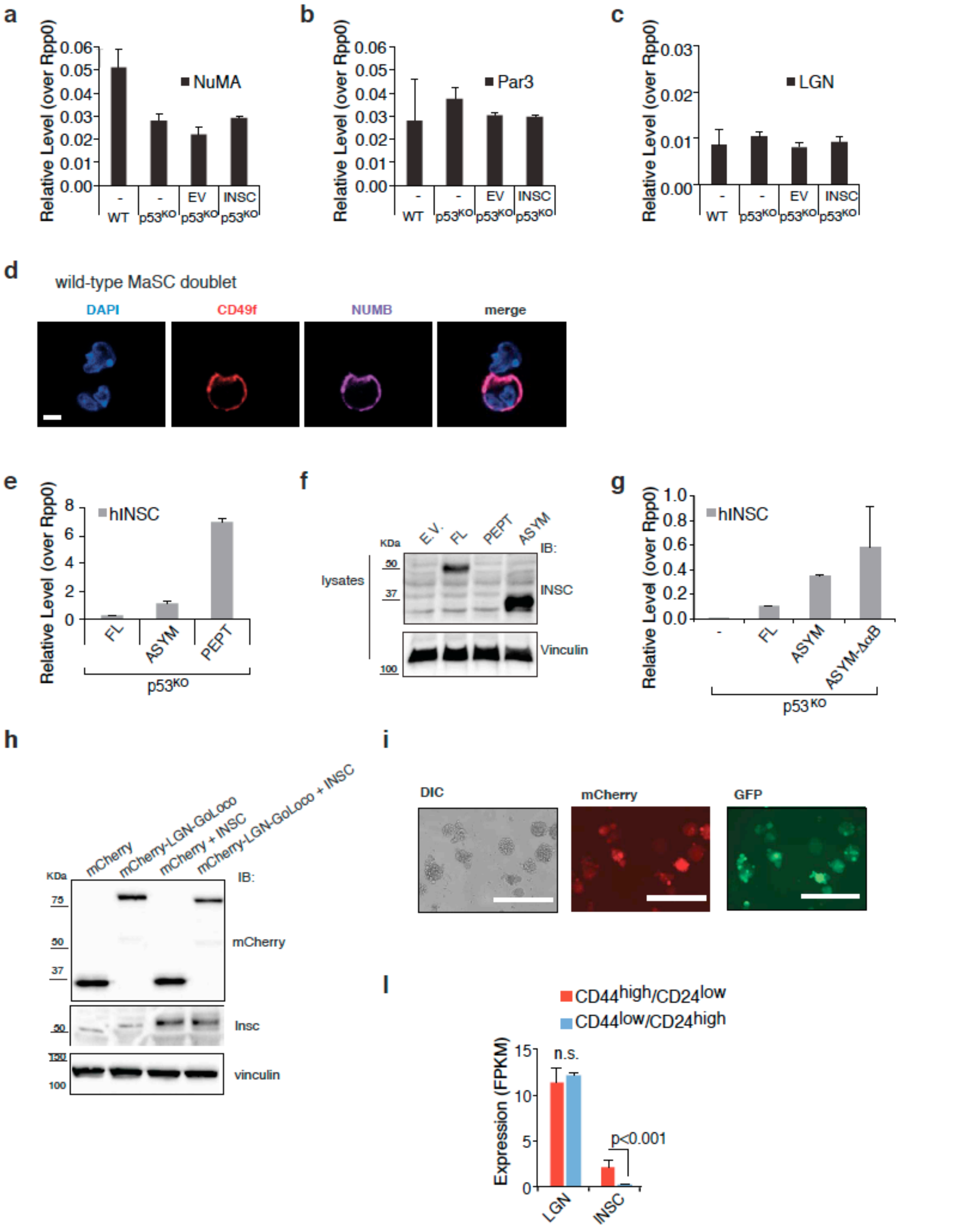


**Supplementary Figure 6. ACDs of p53-null murine MaSCs require tetrameric assemblies of Insc$^{ASYM}$ with Gαi-bound LGN, but not Par3.** (a-**b**-**c**) NuMA, Par3 and LGN transcript levels evaluated by RT-qPCR on mammospheres isolated from wild-type FVB mice, or p53-KO mice, later infected with an empty lentivirus (EV, control) or with a human full-length Insc (INSC) expressing lentivirus. Transcript levels were normalized to the Rpp0 internal control. Bars represent mean ± s.d. of three replicates. **(d)** Representative immunofluorescence images of cultured MaSCs after the first division showing the co-

localization of the basal marker CD49f and the fate determinant Numb. The distribution of Numb and CD49f were used to monitor ACD and to discriminate MaSCs from progenitor daughter cells. DNA is visualized with DAPI. Scale bar, 10 μm. (**e-f**) Transcripts and protein expression levels of Insc constructs (full-length, FL; asymmetric domain, ASYM; and LGN-binding peptide, PEPT) lentivirally tranduced in mammospheres obtained from p53-KO mice evaluated by RT-qPCR and by immunoblot. (**g**) Transcript levels of Insc constructs (full-length, FL; asymmetric domain, ASYM; and ASYM-ΔαB) lentivirally tranduced in mammospheres obtained from p53-KO mice evaluated by RT-qPCR. (**h**) Protein expression levels of Insc full-length (FL) with mCherry and mCherry-LGN-GoLoco lentivirally transduced in mammospheres obtained from p53-KO mice evaluated by immunoblot with the indicated antibodies. SFE assays conducted with these lentivirally transduced mammospheres are presented in Fig. 5j. (**i**) Representative images of mammospheres lentivirally transduced with mCherry and pCDH vectors expressing Insc and a GFP reporter. In each panel, white bars correspond to 400 μm. (**l**) Expression levels of LGN and Insc in HMLE human mammary epithelial cells separated in $CD44^{high}/CD24^{low}$ cells and their non-stem counterpart $CD44^{low}/CD24^{high}$ cells. $CD44^{high}/CD24^{low}$ and $CD44^{low}/CD24^{high}$ cells were isolated by FACS-sorting as previously described[33]. Data shows average and s.e.m. of three independent biological replicates, and are reported as Fragments Per Kilobase of exon per million fragments Mapped (FPKM). The Insc transcript is present only in the stem-like $CD44^{high}/CD24^{low}$ population at levels significantly lower than LGN.

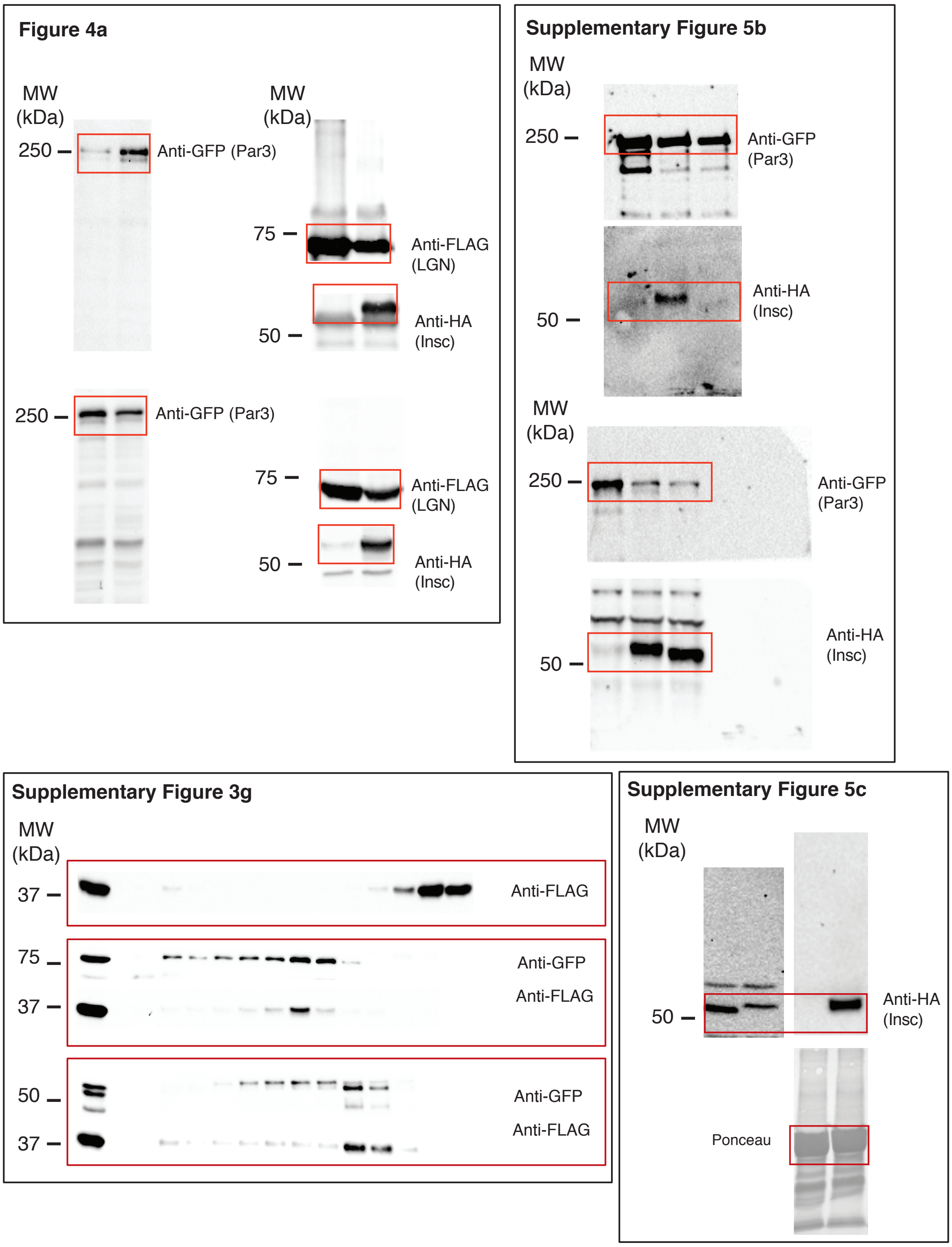


**Supplementary Figure 7. Uncropped images of gels presented in the main and supplementary figures.**

**Supplementary Table 1. SAXS data collection and scattering derived parameters**

| | **Pins$^{TPR}$-Insc$^{ASYM}$** |
|---|---|
| **Data collection** | ESRF-BM29 |
| Wavelength (Å) | 0.99 |
| q-range (Å) | 0.0032 – 0.49 |
| Exposure time (sec) | 2 per frame |
| Temperature (K) | 293 |
| | |
| **Structural parameters** | |
| $I_0$ ($cm^{-1}$) [from P(r)] | 0.2531 ± 0.0004 |
| Rg ($Å^{-1}$) [from P(r)] | 38.74 |
| $I_0$ ($cm^{-1}$) [from Guinier] | 0.2730 ± 0.0002 |
| Rg ($Å^{-1}$) [from Guinier] | 41.1 ± 1.5 |
| Porod volume $V_p$ ($Å^3$) | $318 \cdot 10^3$ |
| Molecular mass $M_r$ [from $V_p$] (kDa) | 187 |
| Calculated monomeric Mr from sequence (kDa) | 172 |
| | |
| **Software employed** | |
| Primary data reduction | BM29 online data analysis, Primus |
| 1D Data processing | Primus, Gnom |
| *Ab initio* analysis | DAMMIF and DAMMIN |

**Supplementary Table 2. Real-Time PCR primers**

| **Primer Name** | Primer sequence |
|---|---|
| mmInsc-for : | TCTACAGTTGAATGCAATCCGTG |
| mmInsc-rev: | GGTCCCGAGTATAAGGGGTGT |
| mmLGN-for: | TTGATAAGCATGAGGGAAGACCA |
| mmLGN-rev: | GGCAGTCCCCTGATTTACATAGA |
| mmPar3-for: | GGAGATGGCCGCATGAAAGTT |
| mmPar3-rev: | CTCCAAGCGATGCACCTGTAT |
| mmNuMA-for: | CCCAAGGGAGGAATAGCTTCT |
| mmNuMA-rev: | CTCTGCGATGCGGTTCCAA |
| mmRpp0-for: | TTCATTGTGGGAGCAGAC |
| mmRpp0-rev: | TTGGTGGTACAAACAGGTATTGA |
| hsINSC-for: | ATGATGGCACTGCCTGGAG |
| hsINSC-rev: | CTGCAGGACACACATGCACT |
