# Supplementary material for: Insc:LGN tetramers promote asymmetric divisions of mammary stem cells
Source: Nat Commun. 2018 Mar 9;9:1025. doi: 10.1038/s41467-018-03343-4 (PMC5844954; doi:10.1038/s41467-018-03343-4)
Supplement: Supplementary file 3 — Description of Additional Supplementary Files [file 41467_2018_3343_MOESM3_ESM.pdf]

Description of Additional Supplementary Files

File Name: Supplementary Movie 1
Description: Animated summary of the close-up views at the interface between subunits of the dLGN-TPR:Insc-ASYM tetramer.

File Name: Supplementary Movie 2
Description: Morphing between the extended and compact conformers of the dLGN-TPR:Insc-ASYM tetramer.
